# Integrated Gut-Heart Axis and Network Pharmacology to Reveal the Mechanisms of the Huoxue Wentong Formula Against Myocardial Ischemia

**DOI:** 10.1155/2022/9538512

**Published:** 2022-05-11

**Authors:** Jianguo Lin, Qingqing Wang, Xin Hua, Jinlong Duan, Kuiwu Yao

**Affiliations:** ^1^Guang'anmen Hospital, China Academy of Chinese Medical Sciences, 100053 Beijing, China; ^2^Tianjin University of Traditional Chinese Medicine, 301617 Tianjin, China; ^3^Shaanxi University of Chinese Medicine, 712046 Xi'an, China; ^4^Eye Hospital China Academy of Chinese Medical Sciences, 100040 Beijing, China

## Abstract

**Background:**

Myocardial ischemia (MI) is a major public health problem with high mortality and morbidity worldwide. Huoxue Wentong formula (HX), a traditional Chinese medicine (TCM) formula, exhibits unambiguous effects on treating MI and preventing cardiovascular diseases. However, the molecular mechanism of the therapeutic effects of HX on MI remains largely unknown.

**Objective:**

This study combined microbiology, metabolomics, and network pharmacology to explore the relationship between the gut microbiota and its metabolites in MI rats and the efficacy of HX.

**Methods:**

First, the MI rat model was established by ligation of left anterior descending. Echocardiography, Masson's staining, and hematoxylin and eosin staining were used to evaluate the effect of HX on MI. Then, fecal metabolomics and 16S rRNA sequencing were used to obtain the microbial and metabolic characteristics of HX on MI. After that, network pharmacology was used to predict the target and action pathway of HX in treating MI. Finally, the relationship between fecal metabolites and target was explored through bioinformatics.

**Results:**

HX can improve the cardiac function and ameliorated myocardial fibrosis in MI rats. Moreover, HX can affect the gut microbiota community and metabolites of MI rats, especially *Bacteroides*, *Deferribacteres*, *Ruminococcus_sp._zagget7*, *Acidobacteria*, daidzein, L-lactic acid, and malate. Network pharmacology found that HX can function through tumor necrosis factor (TNF), tumor protein p53 (TP53), interleukin 6 (IL6), vascular endothelial growth factor A (VEGFA), fos proto-oncogene (FOS), bcl2-associated *X* (BAX), myeloperoxidase (MPO), PI3K-Akt signaling pathways, and HIF-1 signaling pathway. The mechanism study showed that the anti-MI effect of HX was related to valine, leucine, and isoleucine biosynthesis, fatty acid biosynthesis, and arachidonic acid metabolism.

**Conclusion:**

This study demonstrates that HX treated MI rats in a multitarget and multipathway manner. Its mechanism is related to the change of gut microbiota and the regulation of valine, leucine and isoleucine biosynthesis, fatty acid biosynthesis, and arachidonic acid metabolism.

## 1. Introduction

Cardiovascular diseases (CVD) are the leading cause of global mortality; it estimates that the number of CVD deaths increased from 12.1 million in 1990, reaching 18.6 million [1]. Myocardial ischemia (MI) is the largest cause of CVD, causing about 1.7 million deaths in China in 2016, and is the second leading cause of death in the Chinese population [2].

MI is identified as an irreversible myocardial disease which is based on necrotic damage of a myocyte [3]. At present, timely and effective myocardial reperfusion is the main treatment for MI. However, the process of reperfusion may lead to reperfusion-induced arrhythmias, myocardial stunning, microvascular obstruction, and other problems [4, 5].

Based on the theories of traditional Chinese medicine (TCM), MI is equivalent to the term of “Xiong Bi.” TCM has a rich history and experience in the treatment of CVD [6–10]. Huoxue Wentong formula (HX) is a TCM formula formulated by Boshou Xue, a master of TCM, in long-term clinical practice. It is made up of six kinds of Chinese medicinal materials, including *Salvia miltiorrhiza* Bunge, *Ligusticum chuanxiong* Hort, *Spatholobus suberectus* Dunn, *Codonopsis pilosula* (Franch.) Nannf, *Cinnamomum cassia* Presl, and *Paeonia lactiflora* Pall.

HX has shown beneficial effects in the treatment of CVD in our preliminary research studies [11]. Owing to the complexity of the chemical composition of HX, it is difficult to clarify the multicomponent, multitarget, and multipathway mechanism of HX therapy for MI only by traditional experimental methods. It is necessary to use bioinformatics technology to further explain the mechanism of TCM [12–14].

There is growing evidence that gut microbiota-derived processes in general are linked to numerous CVD relevant phenotypes [15–17]. Clinical studies have found that MI-induced intestinal hypoperfusion can cause intestinal mucosal injury and intestinal barrier failure, trigger the translocation of intestinal microorganisms and products into the systemic circulation, activate monocytes, and trigger inflammation [18]. Gut microbiota alters the composition of the immune system and impairs cardiac repair after ischemia [19]. Intestinal metabolites are the basis of the function of gut microbiota [20]. When the internal and external environment changes, the body will cause a series of changes in metabolomics. Metabolomics is rich in information and is one of the most predictive pathways for phenotypes [21]. Therefore, in the present study, integrating microbiome, metabolomics, and network pharmacology analysis was conducted to explore the relationship among HX, gut microbiota, and MI.

## 2. Materials and Methods

### 2.1. Drugs and Reagents

HX were supplied by Sichuan Neo-Green Pharmaceutical Technology Development Co. Ltd (Sichuan, China). HX contains the following ingredients: *Salvia miltiorrhiza* Bunge 30 g, *Codonopsis pilosula* (Franch.) Nannf 10 g*, Paeonia lactiflora* Pall 10 g, *Spatholobus suberectus* Dunn 10 g, *Ligusticum chuanxiong* Hort 9 g, and *Cinnamomum cassia* Presl 10 g. The batch numbers of the abovementioned six Chinese herbal formula granules are 20090036, 20080104, 20020114, 20100087, 20070107, and 20100060, respectively. For all HXWTF herbal materials, distribution of particles, and quality control data, see supplementary Materials (available here).

The following reagents were mainly used: Hematoxylin and Eosin (HE) Staining Kit (C0105S, Beyotime, China), Masson's Trichrome Stain Kit (G1340, Solarbio, China), isoflurane (R510-22-10,RWD, China), paraformaldehyde, 4% (P1110, Solarbio, China), DNeasy PowerSoil Kit (12888, QIAGEN, Germany), QIAamp 96 PowerFecal QIAcube HT Kit (51531, QIAGEN, Germany), Qubit dsDNA Assay Kit (Q32854, Life Technologies, USA), and Tks Gflex DNA polymerase (R060B, Takara, Japan).

### 2.2. Animals

Male Sprague Dawley rats (weighing 190–200 g, 6-7 weeks of age) were obtained from Beijing Huafukang Biotechnology Co. Ltd. was raised in the laboratory animal center of the Guang'anmen Hospital, China Academy of Chinese Medical Sciences. All rats were housed in specific pathogen-free animal rooms. The temperature of the animal room is 20–25°C, and the relative humidity is 55–60%. The rats were fed with standard rat feed and were free to eat and drink water. All rats feeding methods and animal experiment procedures strictly follow the relevant guidelines stipulated by the experimental animal ethics committee of the Guang'anmen Hospital, China Academy of Chinese Medical Sciences.

### 2.3. Model of MI and Drug Treatment

The rats model of MI was established by left anterior descending (LAD) ligation as previously described [22, 23]. Rats were anaesthetized, endotracheally intubated, and mechanically ventilated. Left thoracotomy between the fourth and fifth ribs was performed, and the LAD was ligated. Successful LAD ligation is marked by changes in the electrocardiogram (ECG) consistent with ST-segment elevation and color changes in ischemic areas.

Two days after the operation, the surviving rats were randomly divided into three groups: (1) sham-operated group (Sham, without LAD ligation); (2) model group (Model, LAD ligation); and (3) HX-treated group (HX, LAD ligation, and intragastrical administered HX at 840 mg/kg). The dosage of intragastric administration was the equivalent dose converted by the surface area of the human, and the sham group and model group were given equivalent distilled water. The drugs were administered once a day for 4 weeks.

### 2.4. Echocardiographic Evaluation of the Cardiac Function

After chest fur was removed with depilatory cream, the rats were anaesthetized with isoflurane and fixed on a measuring plate in a supine position. The Vevo 3100 Imaging System (Fujifilm Visual Sonics Vevo 3100, Toronto, Canada) was used to observe and record the echocardiograph of rats. The cardiac function indexes, including ejection fraction (EF), fractional shortening (FS), left ventricular end-diastolic diameter (LVEDD), and left ventricular end-systolic diameter (LVESD) were calculated by the Vevo LAB software.

### 2.5. Histological Analysis

After the rats were euthanized, their hearts were harvested and fixed in 4% paraformaldehyde. After paraffin embedding and slicing, the heart sections were subjected to routine HE staining and Masson's trichrome staining. The Image-Pro Plus 6.0 was used to measure the volume fraction of collagen fibers.

### 2.6. Gut Microbiota Analysis

Stool samples were collected at weeks 3 and then frozen and stored at −80°C until analysis. Total genomic DNA was extracted using DNA Extraction Kit following the manufacturer's instructions. The concentration of DNA was verified with NanoDrop and agarose gel. Using genomic DNA as a template, the 16SV3-V4 region was selected for amplification (343F-5′-TACGGRAGCAGCAG-3′; 798R-5′-AGGGTATCTAATCCT-3′). PCR amplification was performed using the Tks Gflex DNA Polymerase kit. Amplicon quality was visualized using gel electrophoresis, purified with AMPure XP beads (Agencourt), and amplified for another round of PCR. After being purified with the AMPure XP beads again, the final amplicon was quantified using the Qubit dsDNA assay kit. Equal amounts of purified amplicon were pooled for subsequent sequencing.

Raw sequencing data were in the FASTQ format. Paired-end reads were then preprocessed using Trimmomatic software [24] to detect and cut off ambiguous bases (N). After trimming, paired-end reads were assembled using FLASH software [25]. Clean reads were subjected to primer sequence removal and clustering to generate operational taxonomic units (OTUs) using VSEARCH software [26] with a 97% similarity cutoff. The representative read of each OTU was selected using QIIME package [27]. All representative reads were annotated and blasted against Silva database Version 123 (16s/18s rDNA) using a RDP classifier (confidence threshold was 70%). All representative reads were annotated and blasted against Unite database (ITSs rDNA) using blast [28].

### 2.7. Metabolomics Analysis

A stool sample of 60 mg was weighed and GC-MS metabolomics analysis was performed after pretreatment.

#### 2.7.1. Analysis Conditions by GC-MS

1. Chromatographic conditions: A DB-5MS capillary column (30 m × 0.25 mm × 0.25 *μ*m, Agilent J&W Scientific, Folsom, CA, USA) was filled with high purity helium gas as the carrier gas at a flow rate of 1.0 mL/min. The inlet temperature was 260°C. The injection volume was 1 *μ*L, and the solvent delay was 5 min. Programmed temperature: the initial temperature of the column temperature box is 60°C and was maintained for 0.5 min; temperature was programmed at 8°C/min to 125°C; 5°C/min temperature to 210°C; temperature rises to 270°C at 10°C/min; heat up to 305°C at 20°C/min and hold for 5 min.
2. Mass spectrometry conditions: electron bombarded ion source (EI), ion source temperature 230°C, four-stage pole temperature 150°C, electron energy 70 eV. SCAN mode: Full SCAN mode (SCAN), quality SCAN range: M/Z 50–500.
3. AnalysisBaseFileConverter software was used to convert the raw data (.D format) to.abf format, and then the.abf data were imported into the MD-DIAL software for data processing. Data were transformed by log10, and the resulting data matrix was then imported into R ropls package.

### 2.8. Network Pharmacology and Molecular Docking Analysis

The active ingredients and targets of HX were collected using Traditional Chinese Medicine Systems Pharmacology Database and Analysis Platform (TCMSP, https://tcmspw.com) [29]; the TCMSP parameter was set as bioavailability (OB) ≥ 30% and drug-like properties (DL) ≥ 0.18. Disease targets of MI were collected by the GeneCards database (https://www.genecards.org/) [30] and DisGeNET database (https://www.disgenet.org/) [31]. The predictive targets of HX to treat MI were obtained by overlapping these targets. The protein-protein interaction (PPI) network of overlapping targets was constructed using the STRING platform (https://string-db.org/) [32]. BioGPS (http://biogps.org/) was used to obtain the distribution of key targets in human organs and tissues. Metascape database [33] was used to analyze Kyoto Encyclopedia of Genes and Genomes (KEGG). ClueGO was used for Gene Ontology (GO) enrichment. Cytoscape 3.9.0 was used for network topology analysis. MetaboAnalyst (https://www.metaboanalyst.ca/) was used for metabolite analysis.

Key targets and key ingredients were selected for molecular docking. The key target's structure was obtained through the PDB database (https://www.rcsb.org/), the key ingredients structure was obtained through the PubChem database (https://pubchem.ncbi.nlm.nih.gov/).Pymol, and Autodock Vina was used to conduct molecular docking.

### 2.9. Statistical Analysis

Statistical analysis was performed using GraphPad Prism 8 and R 3.6.3. All data are presented as mean ± standard deviation (SD). *T*-test (normal distribution data) and Wilcoxon rank sum test (non-normal distribution data) were used for comparison between two independent groups. One-way analysis of variance (normal distribution data) was used for comparison between multiple independent groups. All statistical analyses are bilateral tests. *p* < 0.05 was considered statistically significant.

## 3. Results

### 3.1. HX-Ameliorated Cardiac Function in MI Rats

**Figure 1:**
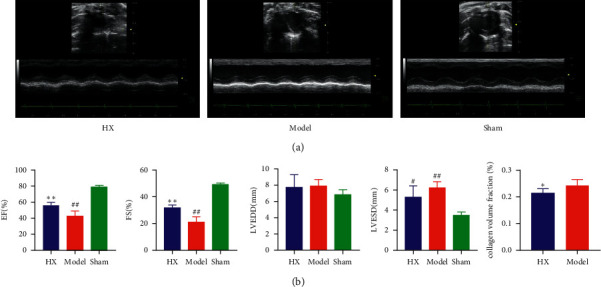
HX improved cardiac function in MI rats. (a) Representative echocardiograms after 4 weeks of HX treatment. (b) Cardiac function indexes. ^#^*p* < 0.05, ^##^*p* < 0.01 vs the sham group; ^*∗*^*p* < 0.05,  ^*∗∗*^*p* < 0.01 vs the model group; *n* = 6.

We evaluated the effect of HX on MI rats after 4 weeks of drug treatment. The results showed that compared with the sham group, EF, FS, and LVESD in the model group were significantly deteriorated. After treating with HX, these cardiac function indices were dramatically improved. The results suggested that HX can improve the cardiac function of MI rats (Figure 1).

### 3.2. HX-Ameliorated Cardiac Fibrosis in MI Rats

**Figure 2:**
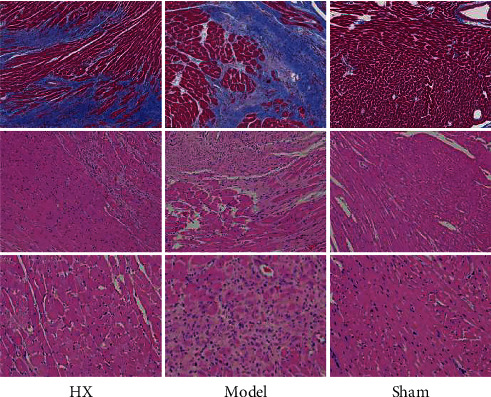
HX improved cardiac fibrosis in MI rats. Masson's staining showed blue collagen fibers and red muscle fibers. HE stained blue nucleus and pink cytoplasm.

We evaluate myocardial fibrosis after MI in rats by Masson's trichromatic staining and HE staining. The results showed that the myocardial structure of the sham group was normal without collagen fiber hyperplasia. The myocardial fibers in the model group were disordered, and there was a large amount of blue collagen fiber deposition. Compared with the model group, blue collagen fiber deposition was significantly reduced in the HX group (Figures 1(b) and 2).

### 3.3. Gut Microbiota Analysis

**Figure 3:**
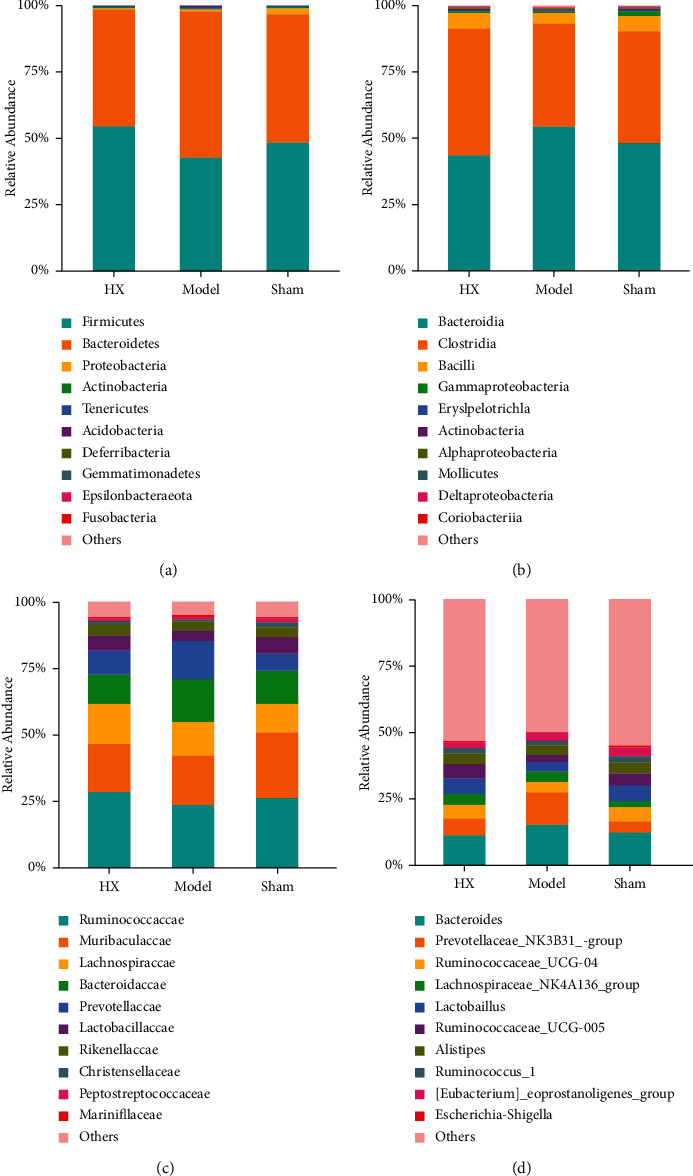
Comparison of the community structure at the phylum, class, family, and genus levels between the groups (a) phylum level; (b) class level; (c) family level; and (d) genus level.

**Figure 4:**
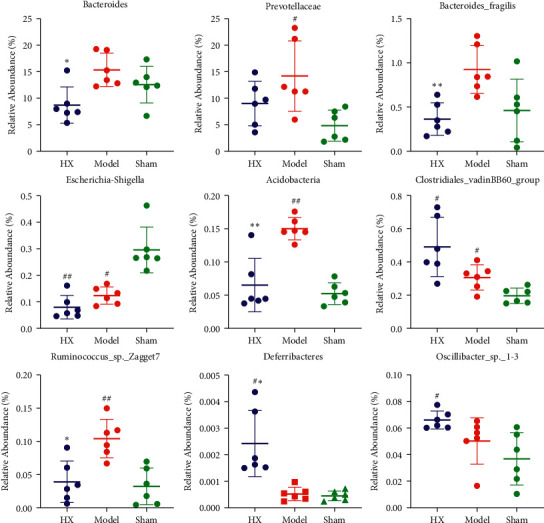
Relative abundance of the differential gut microbiota between the groups. ^#^*p* < 0.05, ^##^*p* < 0.01 vs the sham group; ^*∗*^*p* < 0.05,  ^*∗∗*^*p* < 0.01 vs the model group; *n* = 6.

**Figure 5:**
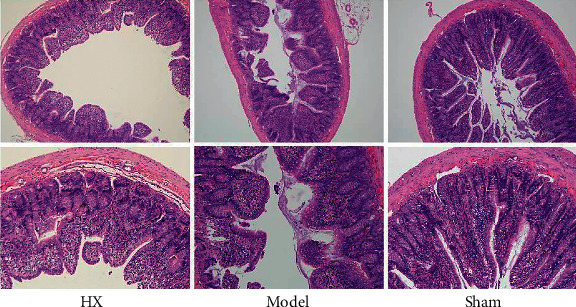
HE staining of the ileum tissue of rats. There was no significant difference in histological characteristics between the groups.

We evaluate gut microbiota after MI in rats by 16S rRNA sequencing. The results showed that *Firmicutes*, *Bacteroidetes, Proteobacteria*, and *Actinobacteria* were the main fecal microbial community groups in the phylum level. Compared with the sham group, the ratio of *Firmicutes*/*Bacteroidete* (F/B) in the model group showed a decreasing trend. Compared with the model group, the F/B value of the HX group showed an increasing trend. Furthermore, from the level of class, family, and genus, there were some differences in the distribution of fecal microbial community in each group (Figure 3). The scatter diagram in the class level showed that compared with the sham group, *Prevotellaceae*, *Actinobacteria*, and *Ruminococcus*_sp._Zagget7 were much abundant in the model group. Compared with the model group, *Bacteroides*, *Bacteroides_fragilis*, *Acidobacteria*, and *Ruminococcus*_sp._Zagget7 in the HX group were significantly reduced. It is worth noting that after treating with HX, *Deferribacteres* significantly increased (Figure 4). In addition, we made HE staining on the ileum of rats. The results showed that the intestinal pathological changes were not significant, and the integrity of ileum was good in the three groups (Figure 5).

### 3.4. Metabolomics Analysis

**Figure 6:**
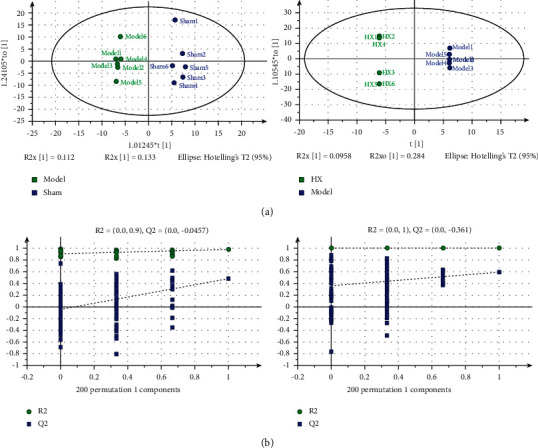
Diagram of metabolite analysis (a) OPLS-DA score plots between the groups. (b) Permutation tests among the groups.

Orthogonal projections to latent structures (OPLS-DA) model clustering is a statistical method of supervised discriminant analysis. Based on PLS-DA, this method can eliminate the information irrelevant to classification, improve the analytical ability and validity of the model, and maximize the differences between different groups within the model. As shown in Figure 6(a), the model group and the sham group were significantly separated in the OPLS-DA diagram, and the sample dispersion degree was good, indicating that the grouping was good and there were differences between groups. The HX group and the model group were obviously separated in the OPLS-DA diagram, indicating that there was an intergroup difference. The model group had a high degree of aggregation, indicating that the samples were stable and reliable. To prevent overfitting of the model, 7-fold cross validation and 200 response permutation testing were used to evaluate the quality of the model. The permutation test results showed that the predictive ability of this model was excellent (Figure 6(b)).

### 3.5. Differential Metabolite Analysis

**Figure 7:**
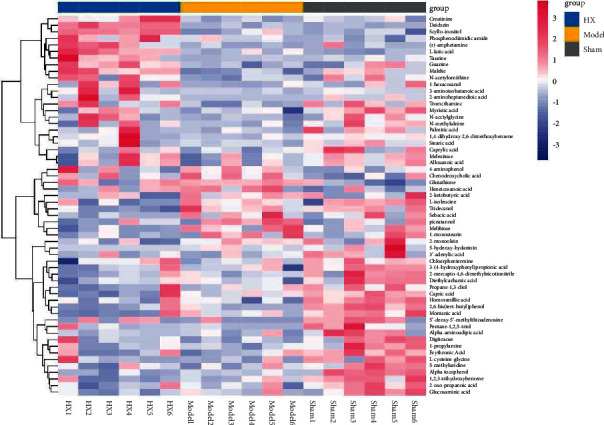
Differential metabolites between the groups. Red represents upregulated metabolites and blue represents downregulated metabolites.

In order to more intuitively display the relationship between samples and the expression differences of metabolites between different samples, we used the OPLS-DA model to screen differential metabolites (screening criteria: VIP > 1, *p* < 0.05). The screening results showed that (Figure 7) the levels of 4-aminophenol, phosphenodiimidic amide, glutathione, heneicosanoic acid, and chenodeoxycholic acid were elevated, and the levels of myristic acid, erythronic acid, 3-aminoisobutanoic acid, N-acetylglycine, caprylic acid etc., were reduced in the model group compared with the sham groups. The levels of daidzein, L-lactic acid, scyllo-inositol, malate, N-acetylornithine, and guanine were elevated, and the levels of 5-hydroxyhydantoin, 1-monostearin, tridecanol, sebacic acid, and melibiose were reduced in the HX group compared with the model groups. The result indicated that HX treatment could change metabolic perturbation.  Table 1 shows the information related to differential metabolites in the HX group and the model group.

### 3.6. Enrichment Analysis of Metabolic Pathways

**Figure 8:**
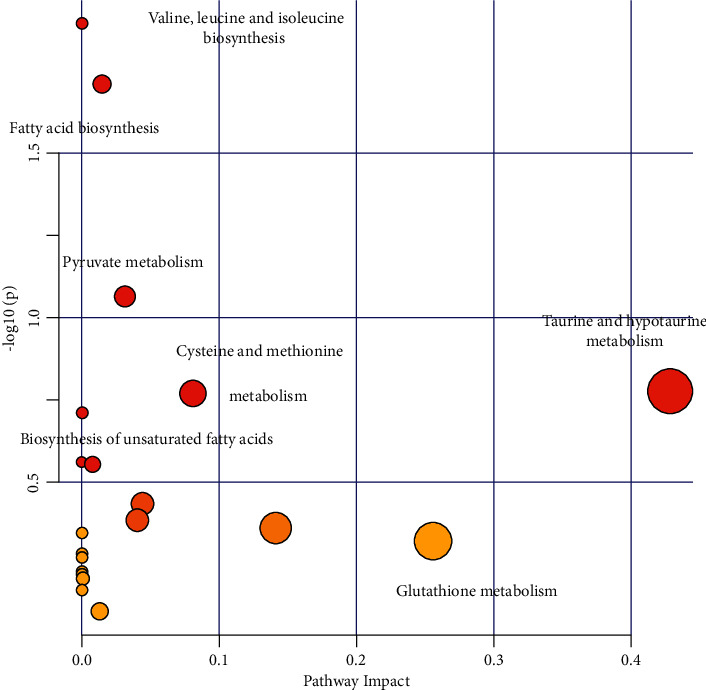
Metabolic pathways analysis, the ordinate represents the metabolite *p* values, and the abscissa represents the pathway impact factor. The lower the *p* values, the darker the red and the larger the circles, which represents a higher pathway impact factor, and redder and larger circles manifest that the pathway is obviously influenced.

To explore the metabolic pathways of HX in MI rats, we imported these differential metabolites to MetaboAnalyst. The result showed that the most influenced metabolic pathways were the aline, leucine, and isoleucine biosynthesis, fatty acid biosynthesis, pyruvate metabolism, taurine and hypotaurine metabolism, and cysteine and methionine metabolism (Figure 8).

### 3.7. Combined Microbiome and Metabonomics Analysis

**Figure 9:**
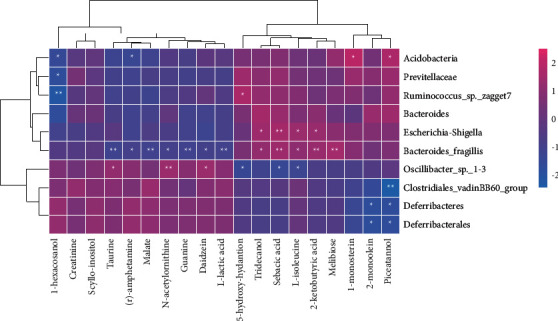
Combined microbiome and metabolite analysis.

To explore the functional relationship between differential gut microbiota changes and differential metabolites after MI, we established two correlation heat maps based on Pearson's correlation coefficient. The result showed that the *Bacteroides_fragilis* was closely related to the upregulation of 2-ketobutyric acid, sebacic acid, and melibiose and the downregulation of taurine, L-isoleucine, malate, and guanine. *Acidobacteria* was closely related to the upregulation of 1-monostearin and piceatannol. *Oscillibacter_sp._1-3* was closely related to the upregulation of taurine, daidzein, and N-acetylornithine and the downregulation of 5-hydroxyhydantoin, L-isoleucine, and sebacic acid. See Figure 9 for details.

### 3.8. Network Pharmacology Analysis

**Figure 10:**
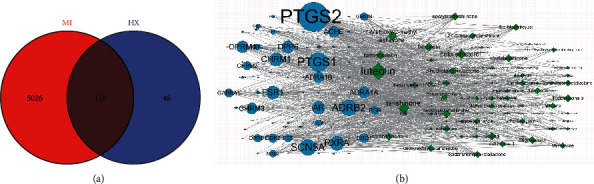
Network pharmacology analysis (a) venn figure of disease and herb (b) compound-target network, the larger the font and size, the greater the degree.

To further explore the mechanisms of HX against MI, we conducted network pharmacology. A total of 158 HX targets were collected through the TCMSP platform. A total of 5136 MI targets were collected through the GeneCards database and DisGeNet database. The intersection of drug targets and disease targets was calculated, and 110 overlapping targets were obtained (Figure 10(a)). Then, Cytoscape was used to construct the compound-target network diagram, and the compounds with a high degree value were obtained (Figure 10(b) and Table 2).

### 3.9. PPI and BioGPS Analysis

**Figure 11:**
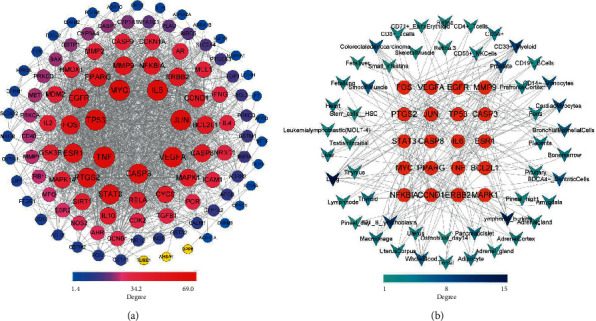
PPI and BioGPS analysis (a) PPI figure of key target. (b) Network map of key target-organ tissues, the node color reflects its degree.

PPI analysis was performed on the overlapping targets. The figure of PPI showed that the key targets were tumor necrosis factor (TNF), tumor protein p53 (TP53), MYC Proto-Oncogene (MYC), interleukin 6 (IL6), vascular endothelial growth factor A (VEGFA), caspase 3 (CASP3), signal transducer and activator of transcription 3 (STAT3), prostaglandin-endoperoxide synthase 2 (PTGS2), estrogen receptor 1 (ESR1), and jun proto-oncogene (JUN) (Figure 11(a)). The BioGPS database was used to analyze the top 20 key targets to obtain the distribution of key targets in human organs and tissues. The key targets are mainly distributed in the lung, lymphoma_burkitts, CD33+_Myeloid, cardiac myocytes, prostate, BDCA4+_dentritic cells, smooth muscles, and whole blood (Figure 11(b)).

### 3.10. Gene Enrichment Analysis

**Figure 12:**
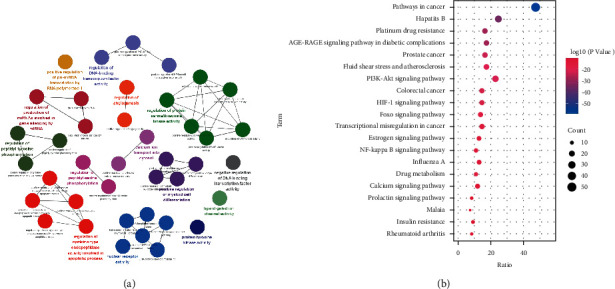
Gene enrichment analysis. (a) Biological process enrichment analysis of key targets by ClueGO. (b) KEGG bubble chart.

To decipher the potential targets of HX treat MI, we performed GO by ClueGO and KEGG pathway enrichment analysis by Metascape. The top terms in biological process (BP) analysis were regulation of angiogenesis, calcium ion transport into cytosol, positive regulation of DNA-binding transcription factor activity, and positive regulation of myeloid cell differentiation (Figure 12(a)). According to the KEGG enrichment analysis, the pathways affected significantly were pathways in cancer, fluid shear stress and atherosclerosis, PI3K-Akt signaling pathway, HIF-1 signaling pathway, and calcium signaling pathway (Figure 12(b)).

### 3.11. Combined Analysis of Metabolomics and Network Pharmacology

**Figure 13:**
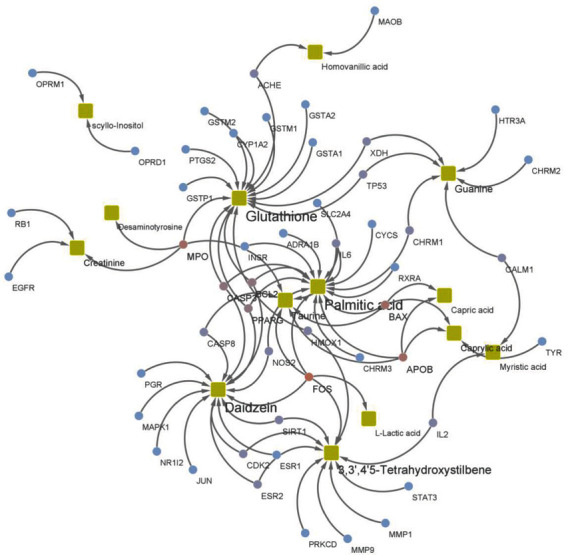
Gene-metabolite interaction network.

To fully understand the mechanism of HX-mediated fecal metabolite therapy for MI, we constructed a gene-metabolite interaction network based differential metabolites and overlapping targets. Moreover, we then found some key targets, including fos proto-oncogene (FOS), bcl2-associated *X* (BAX), myeloperoxidase (MPO), apolipoprotein B (APOB), and peroxisome proliferator-activated receptor gamma (PPARG). The affected pathways were mainly drug metabolism-cytochrome P450, retinol metabolism, and arachidonic acid metabolism (Figure 13).

### 3.12. Molecular Docking Analysis

**Figure 14:**
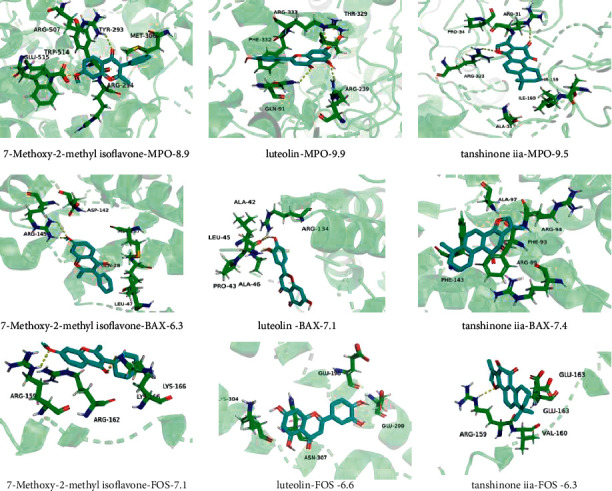
Molecular docking 3D diagram; the number is the binding energy.

To further investigate the possibility of interaction between HX and the key targets, we applied molecular docking studies. We selected luteolin, tanshinone iia, and 7-methoxy-2-methyl isoflavone as binding ligands, selected FOS, BAX, and MPO as molecular docking proteins. The docking analysis showed that the docking energy was ≤−6 kcal·mol^−1^, among which luteolin and MPO have high affinities. Pymol was used to draw the result of molecular docking, in which blue compounds are ligands, green compounds are amino acid residues, and yellow dotted lines are hydrogen bonds. These docking results suggested that HX has a high affinity for key targets and can regulate metabolic processes through these targets (Figure 14).

## 4. Discussion

MI is caused by a variety of pathophysiological factors, involving oxidative stress, calcium overload, immune inflammation, mitochondrial dysfunction, angiogenesis, ion homeostasis, and other processes [34–36]. Many cardiac protection strategies that fail in clinical settings rely on the use of a single targeted approach, but a multitargeted approach targeting multiple intracellular signaling pathways may be a more effective cardiac protection strategy [37, 38]. The human intestine harbors trillions of microbial cells as an essential part of our healthy physiologic ecosystem. Changes in the composition of gut microbiota are associated with the presence of many diseases and/or phenotypes [39]. The gut-heart axis as a novel concept in the fields of CVD research has been recently acknowledged [40, 41]. TCM has a long history. The overall philosophy of TCM is closely related to the core ideas of network pharmacology and network biology, which can systematically overcome complex diseases such as CVD [42, 43]. Thus, it is of great significance to study the treatment of MI with TCM from the perspective of gut microbiota and network pharmacology.

In this study, we selected HX as an intervention Chinese medicine to explore the relationship between its anti-MI efficacy and regulation of MI-related fecal microbiome and metabolome. We found that HX could improve the cardiac function and reduce collagen fiber deposition in MI rats. By 16S rRNA sequencing from rat feces, we found that the rat gut microbiota changed after MI. After taking HX, a part of the altered gut microbiota was reversed. Compared with the other two groups, HX can significantly increase the enrichment of *Deferribacteres*. The study showed that the increased abundance of *Deferribacteres* in the gut microbiota is thought to benefit the regulation of the immune system [44, 45], so we speculated that HX could enhance immunity by improving the abundance of intestinal microbiota. Species of *Bacteroidetes* are ubiquitous symbiotes, accounting for about 30 percent of the human gut microbiome and associated mortality rates of more than 19 percent [46, 47]. The study has shown a significant increase in *Bacteroidetes* in people on a high-fat diet [48]. After taking HX, *Bacteroidetes* were significantly reduced in MI rats. It is speculated that HX may affect CVD by reversing lipid metabolism of *Bacteroides*. At the same time, we also found that *Ruminococcus_sp._*Zagget7 was significantly increased in MI rats after taking HX. There is evidence that rumen *Ruminococcus_sp._*Zagget7 is beneficial bacteria, which has carbohydrate degradation activity and can provide the required nutrients for the host [49]. The microbiome is regulated by diet, and any food we consume may change the microbiome structure in the short term. Therefore, daily use of TCM can change the intestinal homeostasis environment [50].

In order to understand the relationship between gut microbiota metabolites and MI, we screened differential metabolites based on OPLS-DA analysis. The results showed that 19 differential metabolites in the HX group and the model group, and 40 differential metabolites in the model group and the sham group. After taking HX, the contents of daidzein, L-lactic acid, scyllo-inositol, malate, N-acetyl-chlorine, and guanine were significantly increased, which most of the were organic acids and their derivatives. Daidzein is a good antioxidant and anti-inflammatory ingredient [51, 52]; basic experiments showed that daidzein mitigates the progression of diabetic cardiomyopathy by inhibiting NOX-4 induced oxidative stress in cardiac tissues [53]. Malate has a protective effect on CVD and can protect myocardial injury by activating the Nrf2/Keap1 pathway [54]. Subsequently, we made the metabolic pathways of the differential metabolites. The results showed that the differential metabolites were mainly enriched in the valine, leucine and isoleucine biosynthesis and fatty acid biosynthesis. The gut microbiota mainly functions through the short-chain fatty acid pathway [55], and the differential metabolites were enriched in the fatty acid biosynthesis, which provides a scientific basis for speculating that HX may play a role by regulating SCFA metabolism.

We further speculated the pathway of HX action by using network pharmacology. We found that TNF, TP53, MYC, IL6, JUN, VEGFA, CASP3, STAT3, PTGS2, and ESR1 are the key targets of HX therapy for MI. The optimal healing after MI requires timely induction and elimination of inflammation, regulating the release of inflammatory factors such as IL6 and TNF-*α* is of great benefit to myocardial repair [56, 57]. VEGFA is an angiogenic cytokine that promotes angiogenesis in the treatment of ischemic heart disease. It can induce new blood vessel formation and cardiac repair by promoting the release of large amounts of VEGFA by cardiac macrophages through different channels [58, 59]. TP53 is an important tumor suppressor gene, which plays an important role in apoptosis, genomic stability, and inhibition of angiogenesis. Shoffner et al. [60] found Tp53 suppression promotes cardiomyocyte proliferation during heart regeneration. Chen et al. [61] found increased TP53 levels can accelerate heart injury during ischemia reperfusion. Through gene enrichment analysis, we found that these targets were mainly enriched in fluid shear stress and atherosclerosis, PI3K-Akt signaling pathway, HIF-1 signaling pathway, and calcium signaling pathway. Studies have found that ischemic preconditioning can activate the PI3K/Akt signaling pathway in cardiomyocytes, thereby reducing apoptosis, eliminating intracellular reactive oxygen species, and protecting mitochondrial function [62]. Hypoxia is one of the main causes of metabolic changes in MI. HIF and its protein family can reduce cardiac oxygen supply by regulating the oxygen-dependent signal transduction cascade. Activation of HIF-1 signal in endothelial cells can improve the cardiac function after ischemia reperfusion [63, 64].

The target works by producing metabolites. In order to understand the relationship between them, we conducted a joint analysis of metabolites and targets through the Metaboanalyst platform. The results showed that FOS, BAX, and MPO are the main targets. FOS, a gene critical for monocyte and macrophage function, showed that SIRT3-mediated inhibition of FOS through histone H3 deacetylation prevents cardiac fibrosis and inflammation [65]. BAX is a key protein in mitochondria that regulates cell death; the physiological function of BAX ensures tissue homeostasis; dysregulation of BAX leads to aberrant cell death [66]. Studies have shown that inhibition of the BAX expression can reduce myocardial apoptosis after ischemia reperfusion [67, 68]. MPO is a heme peroxidase expressed mainly by neutrophils. Numerous studies have shown a strong association between MPO and CVD, with elevated circulating MPO levels associated with poor prognosis and increased risk of CVD death [69]. MPO produces dysfunctional lipoproteins, which are associated with an increased incidence of atherosclerosis, decreased NO availability, endothelial dysfunction, impaired vascular reactivity, and atherosclerotic plaque instability [70]. Through the above literature research evidence, it is further proved that HX can achieve the effect of treating cardiovascular diseases by regulating these targets. In addition, through the combined analysis of metabolites and targets, we found that the arachidonic acid (AA) pathway plays an important role in the treatment of MI. AA and its derivatives link nutrient metabolism with immunity and inflammation and play a key role in the occurrence and development of cardiovascular diseases. AA can regulate lipid protein, inflammatory response, hemorheology, leukocyte function and platelet activation through cyclooxygenase (COX) pathway, lipoxygenase (LOX) pathway, and cytochrome P450 monooxygenase (CYP) pathway [71–73].

## 5. Conclusions

In summary, the present study demonstrated that HX had a good regulatory effect on MI by echocardiography, HE staining, and Masson's staining. According to microbiome analysis, HX may affect MI by regulating the abundance of *Bacteroides*, *Deferribacteres*, *Ruminococcus*_sp._zagget7, and *Acidobacteria*. Through metabonomics, we found that HX treat MI by changing the metabolism of daidzein, L-lactic acid, scyllo-inositol, malate, guanine, valine, etc. Through network pharmacology, it was revealed that HX mainly regulates TNF, TP53, IL6, VEGFA, FOS, BAX, MPO, and PI3K-Akt signaling pathways, and HIF-1 signaling pathway exerts an anti-MI effect. This study disclosed that the gut microbiota and their metabolites might be involved in the MI and the efficacy of TCM.

## Figures and Tables

**Table 1:** List of significant differential metabolites between the HX and model groups.

Metabolite name	VIP	*p* value
5-Hydroxyhydantoin	4.296961471	0.035549434
Taurine	3.430164964	0.049347795
1-Hexacosanol	3.265528177	0.035544073
(r)-Amphetamine	2.986008432	0.029302733
2-Monoolein	2.690938137	0.021039062
Daidzein	2.624963368	0.008470593
1-Monostearin	2.499821114	0.028543411
L-Isoleucine	1.85895765	0.034322695
Tridecanol	1.8458595	0.004348982
2-Ketobutyric acid	1.808894953	0.043373769
Creatinine	1.807545545	0.047997452
Sebacic acid	1.792625534	0.030546639
Melibiose	1.777031557	0.007011852
L-Lactic acid	1.726303283	0.044068498
Scyllo-inositol	1.638663523	0.042741445
Malate	1.533758785	0.019122454
N-Acetylornithine	1.337941733	0.023766188
Guanine	1.258578618	0.014019157
Piceatannol	1.199917162	0.040843874

**Table 2:** List of the top 10 compounds.

Name	Degree
Luteolin	46
Tanshinone iia	29
Beta-sitosterol	26
7-Methoxy-2-methyl isoflavone	24
Baicalein	23
Formononetin	23
Dihydrotanshinlactone	22
Isotanshinone II	21
Neocryptotanshinone ii	20
Cryptotanshinone	20

## Data Availability

All data used to support the findings of this study are available from the corresponding author upon request.

## Abbreviations

TCM: Traditional Chinese medicine
MI: Myocardial ischemia
HX: Huoxue Wentong formula
CVD: Cardiovascular diseases
ECG: Electrocardiogram
OPLS-DA: Orthogonal projections to latent structures
F/B: *Firmicutes*/*Bacteroidete*
LAD: Left anterior descending
EF: Ejection fraction
FS: Fractional shortening
LVEDD: Left ventricular end-diastolic diameter
LVESD: Leftventricular end-stolic diameter
H&E: Hematoxylin and eosin
PPI: Protein-protein interaction
BP: Biological process
VIP: Variable importance in the projection
TNF: Tumor protein p53
IL6: Interleukin 6
VEGFA: Vascular endothelial growth factor A
CASP3: Caspase 3
STAT3: Signal transducer and activator of transcription 3
PTGS2: Prostaglandin-endoperoxide synthase 2
ESR1: Estrogen receptor
MYC: MYC proto-oncogene
JUN: Jun proto-oncogene
FOS: Fos proto-oncogene
BAX: BCL2-associated X
MPO: Myeloperoxidase
APOB: Apolipoprotein B (APOB)
PPARG: Peroxisome proliferator-activated receptor gamma
KEGG: Kyoto Encyclopedia of Genes and Genomes
GO: Gene Ontology.

## Supplementary Materials

Supplementary Materials This section is about quality report of HX granules.
