# Supplementary material for: Integrated Gut-Heart Axis and Network Pharmacology to Reveal the Mechanisms of the Huoxue Wentong Formula Against Myocardial Ischemia
Source: Evid Based Complement Alternat Med. 2022 May 11;2022:9538512. doi: 10.1155/2022/9538512 (PMC9117028; doi:10.1155/2022/9538512)
Supplement: Supplementary Materials — This section is about quality report of HX granules. [file 9538512.f1.pdf]

Quality report of HXWTF granules

检验报告书

文件编号：XLS-09-MP-0013-OR01(05)

报告书编号： 100104—20020

品　　名	丹参 配方颗粒	规　　格	100g/袋
批　　号	20090036	请验部门	固体制剂车间
代 表 量	8400 袋	收样日期	2020年09月19日
来　　源	包装工序	报告日期	2020年10月09日
检验依据	《丹参配方颗粒企业质量标准》		

检验项目	检验标准	检验结果
性　状	本品应为黄棕色至棕褐色的颗粒；气微，味微苦、涩。	符合规定
薄层鉴别	供试品色谱中，在与丹参对照药材和丹酚酸B对照品色谱相应的位置上，应显相同颜色的荧光斑点。	符合规定
检　查		
粒　度	不能通过一号筛与能通过五号筛的总和应≤15%	1.4%
水　分	≤8.0%	3.5%
溶化性	应全部溶化，允许有轻微浑浊，不得有焦屑等异物	符合规定
装量差异	平均装量应≥100g，每袋装量应≥97g	符合规定
含　量	丹酚酸B≥10mg/g	15mg/g
微生物限度检查		
需氧菌总数	≤10^3 CFU/克（最大可接受2000 CFU/克）	<20 CFU/克
霉菌和酵母菌总数	≤10^2 CFU/克（最大可接受200 CFU/克）	<20 CFU/克
大肠埃希菌	不得检出	未检出

以下空白

四川新绿色药业科技发展有限公司 质检专用章

结论：本品按《丹参配方颗粒企业质量标准》检验，结果符合规定。

批准人：[illegible] 2020.10.09　　复核人：[illegible] 2020.10.09　　编制人：[illegible] 2020.10.09

声明：1. 本报告检验结果仅对送检样品负责；
[illegible]

检 验 报 告 书

文件编号：XLS-09-MP-0013-OR01(05)

报告书编号： 100110—20048

品　　名	党参 配方颗粒	规　　格	100g/袋
批　　号	20080104	请验部门	固体制剂车间
代 表 量	8381 袋	收样日期	2020年08月18日
来　　源	包装工序	报告日期	2020年08月27日
检验依据	《党参配方颗粒企业质量标准》		

检验项目	检验标准	检验结果
性　状	本品应为浅黄色至棕黄色的颗粒；气微，味甜。	符合规定
薄层鉴别	供试品色谱中，在与党参炔苷对照品色谱相应的位置上，应显相同颜色的荧光斑点。	符合规定
检　查		
粒　度	不能通过一号筛与能通过五号筛的总和应≤15%	1.9%
水　分	≤8.0%	3.0%
溶化性	应全部溶化，允许有轻微浑浊，不得有焦屑等异物	符合规定
装量差异	平均装量应≥100g，每袋装量应≥97g	符合规定
浸出物	95%乙醇热浸出物≥10.8%	16.4%
微生物限度检查		
需氧菌总数	≤10^3 CFU/克（最大可接受2000 CFU/克）	20 CFU/克
霉菌和酵母菌总数	≤10^2 CFU/克（最大可接受200 CFU/克）	<10 CFU/克
大肠埃希菌	不得检出	未检出

以下空白

四川新绿色药业科技发展有限公司 质检专用章

结论：本品按《党参配方颗粒企业质量标准》检验，结果符合规定。

批准人：[signature] 2020.08.27　　复核人：[signature] 2020.08.27　　编制人：[signature] 2020.08.27

声明：1、本报告检验结果仅对送检样品负责；
2、对本报告若有异议，应于收到报告之日起七个工作日内向本中心提出，逾期将自动视为承认本检测报告。

检 验 报 告 书

文件编号：XLS-09-MP-0013-OR01(04)

报告书编号： 100070—20013

品　　名	赤芍 配方颗粒	规　　格	100g/袋
批　　号	20020114	请验部门	固体制剂车间
代 表 量	5121 袋	收样日期	2020年02月14日
来　　源	包装工序	报告日期	2020年02月24日
检验依据	《赤芍配方颗粒企业质量标准》		

检验项目	检验标准	检验结果
性　状	本品应为浅棕色至棕褐色的颗粒；气微，味微苦酸涩。	符合规定
薄层鉴别	供试品色谱中，在与赤芍对照药材及芍药苷对照品色谱相应的位置上，应显相同颜色的斑点。	符合规定
检　查		
粒　度	不能通过一号筛与能通过五号筛的总和应≤15%	1.0%
水　分	≤8.0%	2.9%
溶化性	应全部溶化，允许有轻微浑浊，不得有焦屑等异物	符合规定
装量差异	平均装量应≥100g，每袋装量应≥97g	符合规定
含　量	芍药苷≥16.0mg/g	20.1mg/g
微生物限度检查		
需氧菌总数	≤10^3 CFU/克（最大可接受2000 CFU/克）	1.8×10^2 CFU/克
霉菌和酵母菌总数	≤10^2 CFU/克（最大可接受200 CFU/克）	1.3×10^2 CFU/克
大肠埃希菌	不得检出	未检出

以下空白

四川新绿色药业科技发展有限公司 质检专用章

结论：本品按《赤芍配方颗粒企业质量标准》检验，结果符合规定。

负责人：[signature] 2020.02.24　　复核人：[signature] 2020.02.24　　编辑人：[signature] 2020.02.24

检 验 报 告 书

文件编号：XLS-09-MP-0013-OR01(05)

报告书编号： 100084—20039

品　　名	川芎 配方颗粒	规　　格	100g/袋
批　　号	20070107	请验部门	固体制剂车间
代 表 量	2275 袋	收样日期	2020年07月22日
来　　源	包装工序	报告日期	2020年07月31日
检验依据	《川芎配方颗粒企业质量标准》		

检验项目	检验标准	检验结果
性　状	本品应为黄色至黄棕色颗粒；气微香，味微苦、辛。	符合规定
薄层鉴别	供试品色谱中，在与川芎对照药材及阿魏酸对照品色谱相应的位置上，应显相同颜色的荧光斑点。	符合规定
检　查		
粒　度	不能通过一号筛与能通过五号筛的总和应≤15%	2.9%
水　分	≤8.0%	2.9%
溶化性	应全部溶化，允许有轻微浑浊，不得有焦屑等异物	符合规定
装量差异	平均装量应≥100g，每袋装量应≥97g	符合规定
含　量	阿魏酸≥0.3mg/g	1.3mg/g
微生物限度检查		
需氧菌总数	$\leqslant 10^3$ CFU/克（最大可接受2000 CFU/克）	40 CFU/克
霉菌和酵母菌总数	$\leqslant 10^2$ CFU/克（最大可接受200 CFU/克）	10 CFU/克
大肠埃希菌	不得检出	未检出

以下空白

结论：本品按《川芎配方颗粒企业质量标准》检验，结果符合规定。

四川新绿色药业科技发展有限公司 质检专用章

批准人：[illegible] 2020.07.31　　复核人：[illegible] 2020.07.31　　编制人：[illegible] 2020.07.31

声明：1、本报告检验结果仅对送检样品负责；
2、对本报告若有异议，应于收到报告之日起七个工作日内向本中心提出，逾期将自动视为承认本检测报告。

四川新绿色药业科技发展有限公司
Sichuan Neo-green Pharmaceutical Technology Development Co., Ltd.

检验报告书

文件编号：XLS-09-MP-0013-OR01(05)

报告书编号： 100231—20003

品　名	鸡血藤 配方颗粒	规　格	100g/袋
批　号	20100087	请验部门	固体制剂车间
代表量	8742 袋	收样日期	2020年10月20日
来　源	包装工序	报告日期	2020年10月27日
检验依据	《鸡血藤配方颗粒企业质量标准》		

检验项目	检验标准	检验结果
性　状	本品应为浅红棕色至红褐色的颗粒；气微，味涩、微苦。	符合规定
薄层鉴别	供试品色谱中，在与鸡血藤对照药材和芒柄花素对照品色谱相应的位置上，应显相同颜色的荧光斑点。	符合规定
检　查		
粒　度	不能通过一号筛与能通过五号筛的总和应≤15%	2.8%
水　分	≤8.0%	4.0%
溶化性	应全部溶化，允许有轻微浑浊，不得有焦屑等异物	符合规定
装量差异	平均装量应≥100g，每袋装量应≥97g	符合规定
浸出物	95%乙醇热浸出物≥18.0%	22.6%
微生物限度检查		
需氧菌总数	≤10^3 CFU/克（最大可接受2000 CFU/克）	<10 CFU/克
霉菌和酵母菌总数	≤10^2 CFU/克（最大可接受200 CFU/克）	<10 CFU/克
大肠埃希菌	不得检出	未检出

以下空白

（印章：四川新绿色药业科技发展有限公司 质检专用章）

结论：本品按《鸡血藤配方颗粒企业质量标准》检验，结果符合规定。

批准人：［签名］ 2020.10.27　　复核人：［签名］ 2020.10.27　　编制人：［签名］ 2020.10.27

声明：1、本报告检验结果仅对送检样品负责；
2、对本报告若有异议，应于收到报告之日起七个工作日内向本中心提出，逾期将自动视为承认本检测报告。

电话号码： 028-83888888

检测中心地址：四川省彭州市致和镇东河东路279号

四川新绿色药业科技发展有限公司
Sichuan Neo-green Pharmaceutical Technology Development Co., Ltd.

检 验 报 告 书

文件编号：XLS-09-MP-0013-OR01(05)

报告书编号： 100182—20011

品　　名	桂枝 配方颗粒	规　　格	100g/袋
批　　号	20100060	请验部门	固体制剂车间
代 表 量	5916 袋	收样日期	2020年10月10日
来　　源	包装工序	报告日期	2020年10月19日
检验依据	《桂枝配方颗粒企业质量标准》		

检验项目	检验标准	检验结果
性　状	本品应为棕色至棕红色颗粒，气香特异，味甜微辛。	符合规定
薄层鉴别	供试品色谱中，在与桂枝对照药材色谱相应位置上，应显相同颜色的斑点。	符合规定
检　查		
粒　度	不能通过一号筛与能通过五号筛的总和应≤15%	3.6%
水　分	≤8.0%	2.6%
溶化性	应全部溶化，允许有轻微浑浊，不得有焦屑等异物	符合规定
装量差异	平均装量应≥100g，每袋装量应≥97g	符合规定
浸出物	95%乙醇热浸出物≥6.0%	13%
微生物限度检查		
需氧菌总数	≤10^3 CFU/克（最大可接受2000 CFU/克）	10 CFU/克
霉菌和酵母菌总数	≤10^2 CFU/克（最大可接受200 CFU/克）	<10 CFU/克
大肠埃希菌	不得检出	未检出

以下空白

结论：本品按《桂枝配方颗粒企业质量标准》检验，结果符合规定。

四川新绿色药业科技发展有限公司 质检专用章

批准人：[illegible] 2020.10.19　　复核人：[illegible] 2020.10.19　　编制人：[illegible] 2020.10.19

声明：1、本报告检验结果仅对送检样品负责；
2、对本报告若有异议，应于收到报告之日起七个工作日内向本中心提出，逾期将自动视为承认本检测报告。
